# Apatinib promotes autophagy and apoptosis through VEGFR2/STAT3/BCL-2 signaling in osteosarcoma

**DOI:** 10.1038/cddis.2017.422

**Published:** 2017-08-24

**Authors:** Kuisheng Liu, Tingting Ren, Yi Huang, Kunkun Sun, Xing Bao, Shidong Wang, Bingxin Zheng, Wei Guo

**Affiliations:** 1 Musculoskeletal Tumor Center, Peking University People’s Hospital, Beijing, People’s Republic of China; 2 Beijing Key Laboratory of Musculoskeletal Tumor, Beijing, People’s Republic of China; 3 Department of Pathology, Peking University People’s Hospital, Beijing, People’s Republic of China

## Abstract

The cure rate of osteosarcoma has not improved in the past 30 years. The search for new treatments and drugs is urgently needed. Apatinib is a high selectivity inhibitor of vascular endothelial growth factor receptor-2 (VEGFR2) tyrosine kinase, exerting promising antitumoral effect in various tumors. The antitumor effect of Apatinib in human osteosarcoma has never been reported. We investigated the effects of Apatinib in osteosarcoma *in vitro* and *in vivo*. Osteosarcoma patients with high levels of VEGFR2 have poor prognosis. Apatinib can inhibit cell growth of osteosarcoma cells. In addition to cycle arrest and apoptosis, Apatinib induces autophagy. Interestingly, inhibition of autophagy increased Apatinib-induced apoptosis in osteosarcoma cells. Immunoprecipitation confirmed direct binding between VEGFR2 and signal transducer and activator of transcription 3 (STAT3). Downregulation of VEGFR2 by siRNA resulted in STAT3 inhibition in KHOS cells. VEGFR2 and STAT3 are inhibited by Apatinib in KHOS cells, and STAT3 act downstream of VEGFR2. STAT3 and BCL-2 were downregulated by Apatinib. STAT3 knockdown by siRNA reinforced autophagy and apoptosis induced by Apatinib. BCL-2 inhibits autophagy and was apoptosis restrained by Apatinib too. Overexpression of BCL-2 decreased Apatinib-induced apoptosis and autophagy. Apatinib repressed the expression of STAT3 and BCL-2 and suppressed the growth of osteosarcoma *in vivo*. To sum up, deactivation of VEGFR2/STAT3/BCL-2 signal pathway leads to Apatinib-induced growth inhibition of osteosarcoma.

Osteosarcoma is the most common malignant bone and soft tissue tumor that occurs in children and adolescents with a high tendency of local invasion and early systematic metastases.^1, 2^ Because of the new adjuvant chemotherapy and improvement in surgery technology, the 5-year survival rate of patients has improved to approximate 70%.^3^ Unfortunately, the cure rate has not improved in the past 30 years. Therefore, continuing to study new treatments and drugs is urgently needed.

Apatinib (YN968D1) is a novel and high selectivity inhibitor of the vascular endothelial growth factor receptor-2 (VEGFR2) tyrosine kinase, which will block the downstream signal transduction of VEGFR2 at the cellular level.^4^ Apatinib exerts promising antitumoral effect in various tumors.^5, 6, 7^ As shown in a third-phase clinical trial, Apatinib has been proven to be a safe and effective drug in patients with advanced gastric cancer.^8^ However, the antitumoral effect of Apatinib in human osteosarcoma has never been reported.

As a point of convergence for many oncogenic signaling pathways, the transcription factor signal transducer and activator of transcription 3 (STAT3) is involved in cell growth through the downstream signaling molecules such as BCL-2 and cyclin D1.^9, 10, 11^ Recent studies have declared that STAT3 is activated in many tumors such as breast cancer, ovarian cancer, lung cancer and so on.^12, 13, 14^ STAT3 has become a promising target of cancer treatment.^15^

In our research, we evaluated the effect of Apatinib in human osteosarcoma *in vitro* and *in vivo*. In particular, we examined the interaction between apoptosis and autophagy induced by Apatinib.

## Results

### VEGFR2 expression elevated in osteosarcoma and associated with poor prognosis

**Figure 1:**
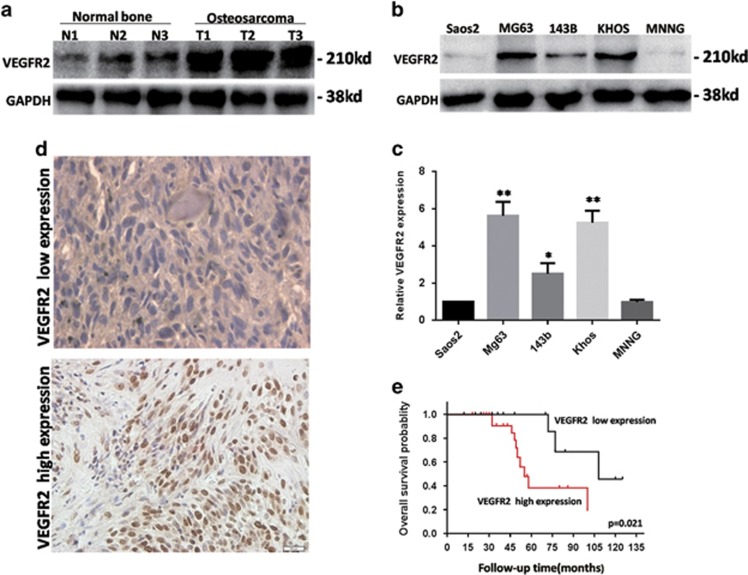
VEGFR2 expression was elevated in osteosarcoma and associated with poor prognosis. (**a**) Western blot showing that VEGFR2 is expressed much higher in osteosarcoma than in normal bone. Glyceraldehyde 3-phosphate dehydrogenase (GAPDH) was used as a control. (**b**) The level of VEGFR2 in osteosarcoma cells was tested with western blot. GAPDH was used as a control. (**c**) The expression of VEGFR2 in osteosarcoma cell lines was detected with reverse transcription-polymerase chain reaction (RT-PCR). *P<0.05,**P<0.01. (**d**) Immunohistochemistry (IHC) staining of VEGFR2 in osteosarcoma samples (× 400). (**e**) Kaplan–Meier curves showed the VEGFR2 expression on overall survival in 45 osteosarcoma patients. These experiments were repeated three times

The expression of VEGFR2 was tested in 15 osteosarcoma tissues and 15 normal bone tissues using western blot analysis. VEGFR2 expression was obviously higher in osteosarcoma tissues than in normal bone tissues. The mRNA and protein levels of VEGFR2 were examined in five osteosarcoma cell lines; KHOS and MG63 cell lines showed a higher level than the other three cell lines (Figures 1b and c). These two cell lines were used for further experiments.

To examine the relationship between VEGFR2 expression and the prognosis in osteosarcoma, immunohistochemistry of VEGFR2 was implemented in 45 osteosarcoma samples, and the results were divided into a high and low expression groups according to the proportion of positive cells and staining intensity (Figure 1d). VEGFR2 expression was detected in the nucleus and cytoplasm. The expression of VEGFR2 was associated with the Enneking stage (*P*=0.019, Table 1).The correlation between the prognosis and VEGFR2 level was further detected. High level of VEGFR2 was related to short overall survival time (*P*=0.021) (Figure 1e). These data confirmed that osteosarcoma patients with a high level of VEGFR2 have a poor prognosis.

### Apatinib suppressed growth of osteosarcoma cells

**Figure 2:**
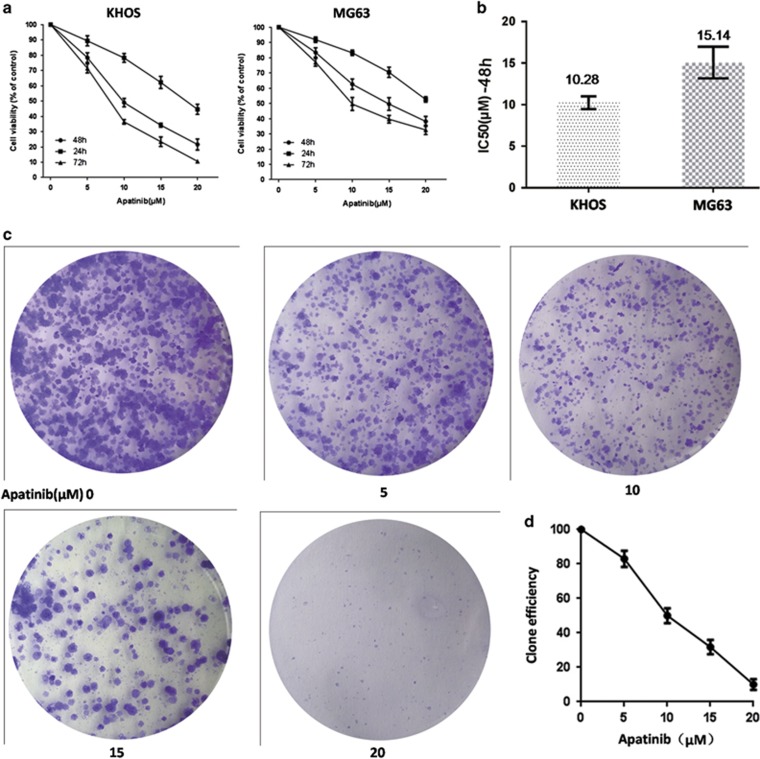
Apatinib inhibited the growth of human osteosarcoma cells. (**a**) Osteosarcoma cells were incubated by Apatinib at various concentrations for 24, 48 and 72 h. Cell viability was detected by CCK8. (**b**) The values of IC50 of Apatinib for 48 h in KHOS and MG63 cells. (**c** and **d**) Colony formation assay of KHOS cells reduced in Apatinib treatment. Each experiment was performed in triplicate

To examine the effects of Apatinib in growth of osteosarcoma cells, we used the KHOS and MG63 cell lines. We cultivated the cell lines in five concentrations of Apatinib for 24, 48 and 72 h, and the cell viability was tested using CCK8. The growth of osteosarcoma cells was suppressed by Apatinib in a time- and dose-dependent manner (Figure 2a). After cultivating with Apatinib for 48 h, the IC50 of Apatinib in KHOS and MG63 cells is shown in Figure 2b, which was used for further experiments. In the colony formation assay, Apatinib reduced colony formation when compared with the negative group (Figures 2c and d). The results suggested that Apatinib suppressed the proliferation of osteosarcoma cells *in vitro*.

### Apatinib induces apoptosis and cell-cycle arrest

**Figure 3:**
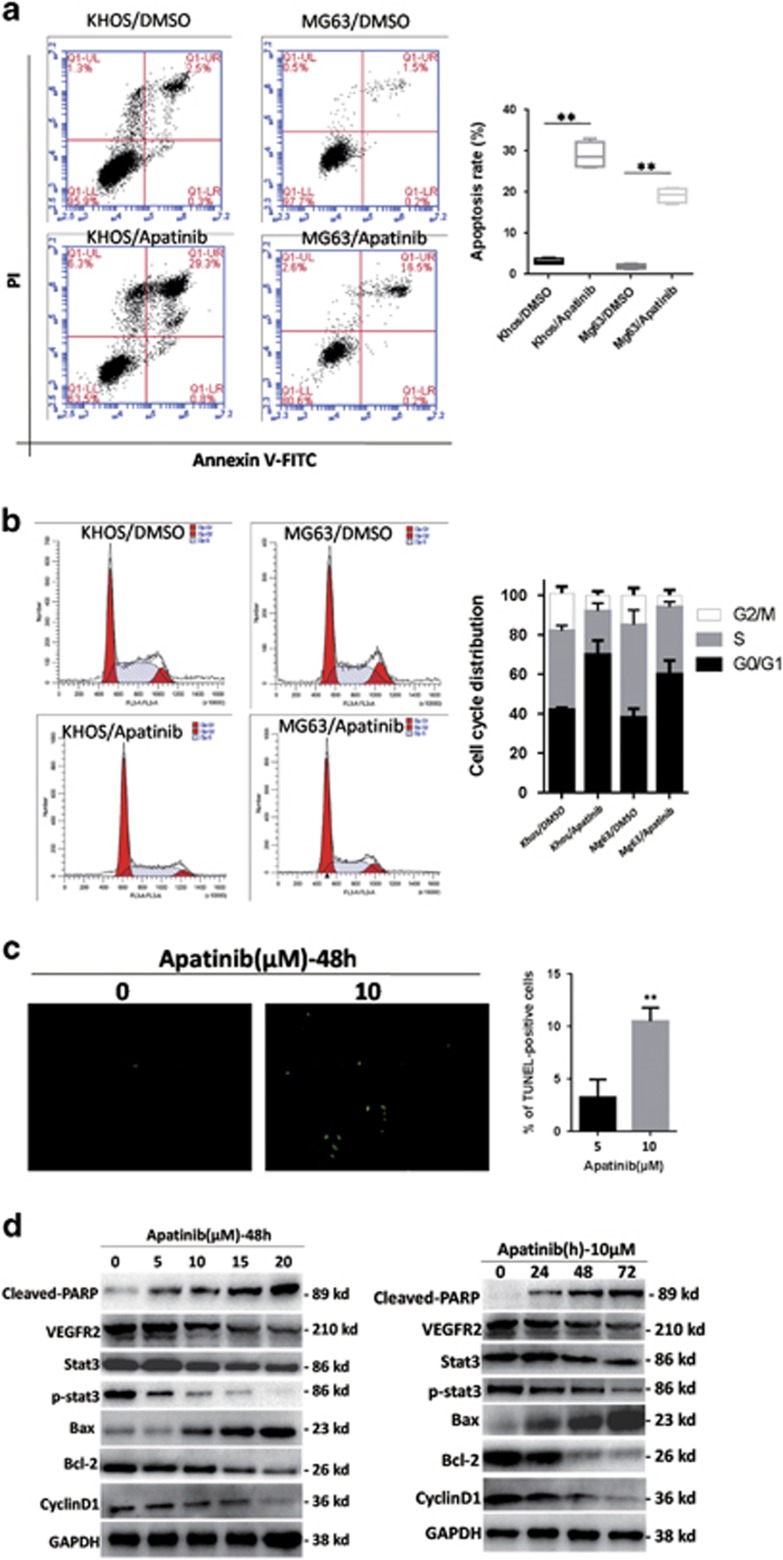
Apatinib-induced apoptosis and cell-cycle arrest. (**a**) Apatinib-induced apoptosis in osteosarcoma cells. The apoptoses were detected by Annexin V-FITC and propidium iodide (PI) staining. **P<0.01. (**b**) Apatinib causes G0/G1 cell-cycle arrest in osteosarcoma cells. Apatinib or dimethyl sulfoxide (DMSO) was added to the culture medium of KHOS and MG63 cells. Cell-cycle distribution was performed after 70% ethanol fixing and stained by PI. (**c**) TUNEL images of KHOS cells after 48 h of Apatinib incubation. The cells labeled with green were positive (× 200). The right graph plotted the ratio of positive cells. **P<0.01. (**d**) The expressions of phase and apoptosis proteins were tested with western blot. Glyceraldehyde 3-phosphate dehydrogenase (GAPDH) was used as a control. These experiments were repeated three times

To evaluate the role of Apatinib in osteosarcoma cells, flow cytometry was used to analyze the cells after Annexin V-FITC and propidium iodide (PI) staining. Apatinib-induced apoptosis significantly when compared with the control group (Figure 3a). As a key indicator of apoptosis, the level of cleaved-PARP increased after treatment with Apatinib for 48 h, or with 10 *μ*M Apatinib for different time points (Figure 3d).

To determine whether Apatinib inhibited cell proliferation by inducing cell-cycle arrest, we evaluated the distribution of cell cycle in osteosarcoma cells treated with Apatinib. As shown in Figure 3b, accumulation of cells by Apatinib resulted in the G0/G1 phase, whereas a corresponding reduction in both KHOS and MG63 cells in the G2/M and S phases. To elucidate the mechanisms we measured the expressions of cyclin D1, a G0/G1-phase-related protein. The expressions of cyclin D1 decreased after treatment with Apatinib, as analyzed by western blot (Figure 3d). Terminal deoxynucleotidyl transferase-mediated nick-end labeling staining (TUNEL staining) were used to confirm apoptosis. Treatment with Apatinib increased TUNEL-positive cells when compared with the control (Figure 3c). All the data suggest that Apatinib induces apoptosis and G0/G1-phase arrest.

### Apatinib-induced human osteosarcoma cell autophagy

**Figure 4:**
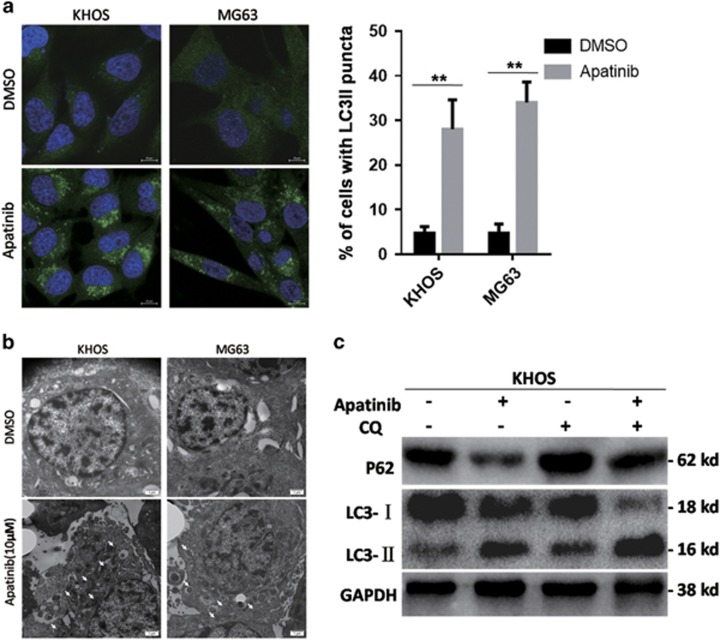
Apatinib-induced autophagy. (**a**) KHOS and MG63 cells growing on coverslips were treated with Apatinib for 48 h. Cells were observed by confocal microscopy. The graph with % of cells with LC3-II puncta was on the right side. **P<0.01. (**b**) The images of TEM: autophagic vacuoles (white arrows) are shown in Apatinib-incubated KHOS and MG63 cells for 48 h. Few autophagic vacuoles were shown in the control group. (**c**) The p62 expression decreased and LC3-II expression increased in the Apatinib group. KHOS cells were incubated by 10 *μ*M Apatinib for 48 h with or without CQ, and markers were treated by SDS-PAGE with GAPDH as the control. These experiments were repeated three times

Autophagy can be a survival mechanism, although it can induce cell death too.^16^ We conducted the following experiments to test whether Apatinib caused autophagy. Autophagy was identified using transmission electron microscopy (TEM). Incubation of KHOS and MG63 cells with Apatinib for 48 h revealed autophagic vacuoles with a unique double membrane, although there were few autophagic vacuoles in the control group (Figure 4b). LC3 is a specific protein in the initial stages of autophagy, and LC3-I was converted to LC3-II in the process of autophagy. As a result, the level of LC3-II immunofluorescence is regarded as a way to seek changes in autophagosomes of the living cells. Apatinib-treated cells displayed a dot pattern of LC3-II fluorescence (Figure 4a), indicating an increase in LC3-II cells in autophagosomes. An increase in LC3-II expression and a decrease in p62 expression were significantly detected in Apatinib-treated KHOS cells by western blot. In addition, when combined with chloroquine (CQ), an inhibitor of the degradation step of autophagy, the expression of LC3-II enhanced when compared with Apatinib alone (Figure 4c). In brief, our data suggest that Apatinib caused autophagy in osteosarcoma cells.

### Inhibition of autophagy sensitized osteosarcoma cells to Apatinib-induced apoptosis

**Figure 5:**
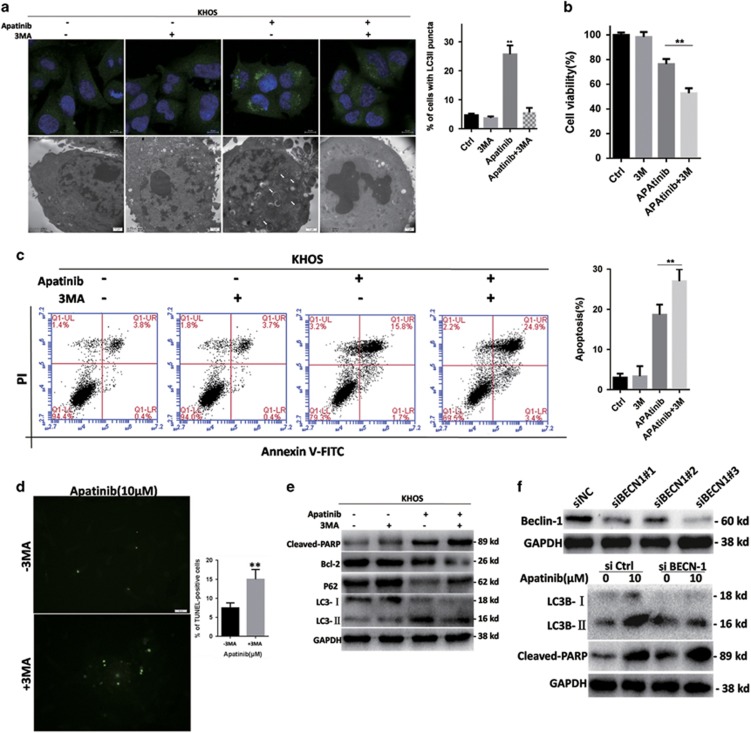
Suppression of autophagy sensitized osteosarcoma cells to Apatinib-induced apoptosis. (**a**) Representative images of confocal microscopy and TEM after Apatinib incubation without or with 3-MA for 48 h. (**b**) Suppression of autophagy with 3-MA decreased the viability of Apatinib-treated cells. (**c**) Suppression of autophagy with 3-MA increased Apatinib-treated cells apoptosis. (**d**) TUNEL staining of KHOS cells after Apatinib incubation without or with 3-MA for 48 h (× 400). (**e**) The expressions of apoptosis- and autophagy-related protein were tested with western blot after Apatinib incubation without or with 3-MA for 48 h. (**f**) Effects of BECN1 knockdown in Apatinib-induced apoptosis. Cleaved-PARP and LC3 were detected by western blot. The data were expressed as mean±SD. **P<0.01. These experiments were repeated three times

Autophagy can inhibit or support cell growth in different cell microenvironments.^17, 18^ The regulation of autophagy may improve the curative effect of cancer therapy, we are eager to know if Apatinib-triggered autophagy in osteosarcoma handed cell death or cell survival. The autophagy inhibitor 3-methyladenine (3-MA) that can inhibit autophagy before the formation of autophagosome was used. As shown in Figure 5a, LC3-II fluorescence punctate pattern weakened and typical autophagic vacuoles decreased, indicating inhibition of autophagy. Pre-treatment by 3-MA obviously decreased the viability of Apatinib-treated cells (Figure 5b) and the ratio of apoptosis cells increased (Figure 5c). TUNEL staining of Apatinib-treated cells significantly reinforced when pre-treated with 3-MA (Figure 5d). Pre-treatment with 3-MA significantly reduced LC3-II and BCL-2, whereas cleaved-PARP and p62 expression increased, indicating a rising apoptosis process, and autophagy inhibited when compared with Apatinib treatment alone. To confirm the cytoprotection of autophagy, the effect of Apatinib was detected in BECN1 cells that were downregulated by siRNA. KHOS cells that transfected with BECN1 siRNA presented LC3-II expression decrease after Apatinib settlement when compared with siRNA negative control, showing that the participation of BECN1 triggered autophagy in osteosarcoma cells. In accordance with 3-MA, knockout of BECN1 with siRNA increased the expression of cleaved-PARP, a significant apoptosis indicator (Figure 5f), suggesting autophagy is a kind of cytoprotection for Apatinib-induced apoptosis. In other words, Apatinib-induced autophagy in osteosarcoma cells, and apoptosis increased when autophagy was inhibited, indicating that autophagy is a protective effect of osteosarcoma cells under the circumstance of Apatinib-induced apoptosis.

### Apatinib suppressed STAT3/BCL-2 signal path

**Figure 6:**
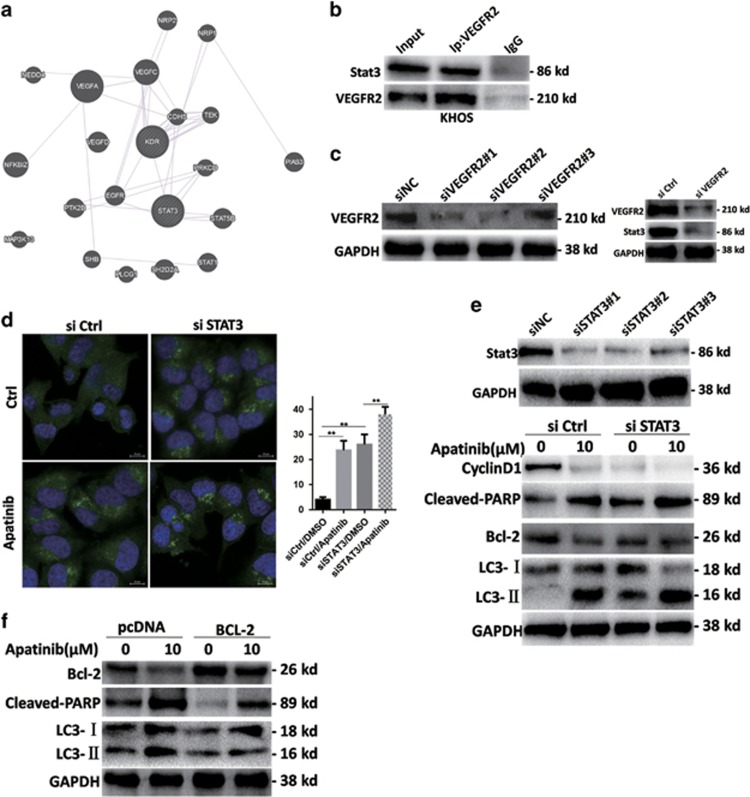
Inactivation of STAT3 enhanced Apatinib effects. (**a**) Bioinformatics predicts interaction between VEGFR2 and STAT3 (http://genemania.org/). (**b**) Immunoprecipitation was used to evaluate the interaction between VEGFR2 and STAT3. (**c**) The level of VEGFR2 and STAT3 was assayed by western blot in VEGFR2 small interfering RNA (siRNA) KHOS cells. (**d**) Typical images of LC3 dot patterns in STAT3 siRNA and negative control siRNA KHOS cells after Apatinib incubation. **P<0.01. (**e**) Western blot was used to evaluate cyclin D1, BCL-2, cleaved-PARP and autophagy-related markers in STAT3 siRNA and negative control siRNA KHOS cells added with Apatinib. (**f**) Overexpression of BCL-2 decreased Apatinib-induced apoptosis and autophagy, and the level of related marker was tested by western blot. These experiments were repeated three times

Bioinformatics prediction shows that there may be an interaction between VEGFR2 and STAT3 (Figure 6a). We further confirmed the interaction between VEGFR2 and STAT3. The antibody against VEGFR2 was able to pull down STAT3 in KHOS cell lines by immunoprecipitation (Figure 6b), confirming direct binding between VEGFR2 and STAT3. Downregulation of VEGFR2 by siRNA resulted in STAT3 inhibition in KHOS cells (Figure 6c). Apatinib, a high selectivity inhibitor of VEGFR2, not only decreased the expression of VEGFR2 but also inhibited the p-STAT3 (Figure 3d). Taken together, VEGFR2 and STAT3 are inhibited by apatinib in KHOS cells and STAT3 is downstream of VEGFR2.

The STAT3 signal path is stimulated regularly in different kinds of tumors.^19, 20, 21, 22^ STAT3 is an important agent for cancer treatment, thus we investigated whether STAT3 plays a role in Apatinib-treated osteosarcoma cells. The expression of phosphorylated STAT3 was significantly inhibited after Apatinib treatment (Figure 3d), as assayed by the western blot analysis. Cyclin D1, a target of STAT3, exerted the same trend (Figure 3d). The STAT3 signaling pathway is involved in Apatinib-treated osteosarcoma cells.

To further confirm the negative regulation of STAT3 signal in Apatinib-treated osteosarcoma cells, we focused on the effect of Apatinib in osteosarcoma cells and how STAT3 was suppressed by siRNA. Consistent with the Apatinib treatment, downregulation of STAT3 caused cell apoptosis, in accordance with the increase of cleaved-PARP (Figure 6e), as well as led to autophagy, increased expression of LC3-II, enhanced Beclin-1 expression and punctate pattern of LC3-II fluorescence (Figures 6d and e). Therefore, these results indicated that STAT3 deactivation was relevant in Apatinib-induced inhibition of cell proliferation, inducing apoptosis and autophagy.

Next, we explored the potential mechanism that STAT3 deactivation by Apatinib induces autophagy and apoptosis in Apatinib-treated cells. It is worth noting that BCL-2 was suppressed by Apatinib (Figure 3d), although the expression of BCL-2 was still further inhibited by Apatinib in STAT3-knockdown cells (Figure 6e). BCL-2 has a significant role in regulating apoptosis and autophagy. KHOS cells that were transfected by BCL-2 overexpression plasmid suppressed Apatinib-induced increase of cleaved-PARP (Figure 6f), indicating that the ectopic expression of BCL-2 can decrease Apatinib-induced apoptosis. Likewise, the ectopic expression of BCL-2 weakened Apatinib-induced autophagy, demonstrated by LC3-II decrease (Figure 6f). The results revealed that Apatinib caused autophagy and apoptosis by way of suppression of STAT3 and inhibition of BCL-2.

### Apatinib inhibited growth of osteosarcoma *in vivo*

**Figure 7:**
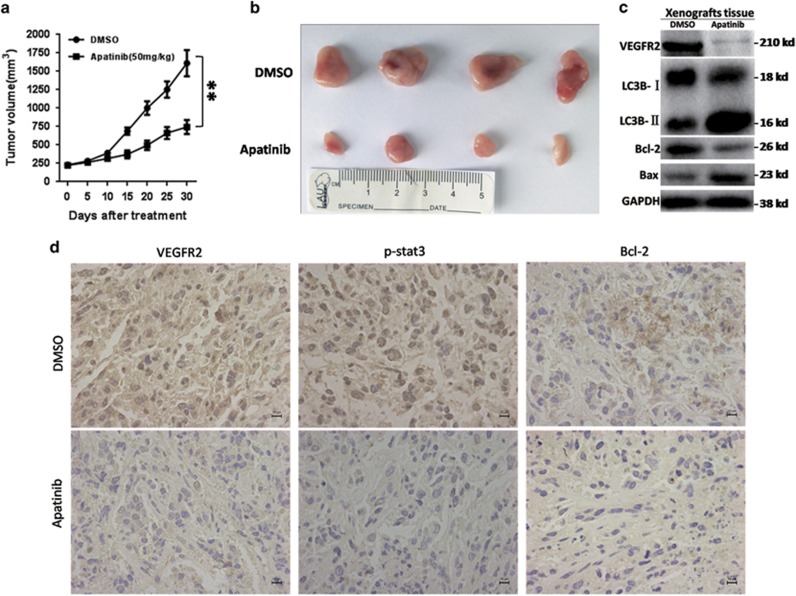
Effects of Apatinib in the growth of osteosarcoma cells *in vivo*. (**a** and **b**) The tumor volume was scaled every 5 days. The xenograft tumors were separated when the animals were killed. Results were represented as mean±S.D. **P<0.01. (**c**) The expressions of VEGFR2, BCL-2, Bax and LC3 of xenograft tumors were tested by western blot. (**d**) Representative immunohistochemistry (IHC) images of VEGFR2, BCL-2 and p-STAT3 of the tumors. These experiments were repeated three times

Apatinib was valid in tumor growth inhibition *in vivo*. The tumor volume decreased when compared with the control group (Figures 7a and b). In accordance with *in vitro* experiment, Figure 7c shows that Apatinib treatment increased the level of LC3-II and Bax, whereas the level of BCL-2 and VEGFR2 decreased *in vivo*. Immunohistochemistry showed that Apatinib decreased the expression of VEGFR2, p-STAT3 and BCL-2 in tumors formed by KHOS cells (Figure 7d). All the results revealed that Apatinib inhibited the growth of osteosarcoma *in vivo*.

## Discussion

Apatinib is a highly selective tyrosine kinase inhibitor to VEGFR2, which exerts promising antitumoral effect in various tumors.^23, 24^ This research demonstrates the antitumoral effects of Apatinib on osteosarcoma cells *in vitro* and *in vivo*. A new mechanism indicates that Apatinib can inactivate STAT3 mediated by VEGFR2 and suppress the BCL-2, resulting in autophagy and apoptosis.

Cell apoptosis and autophagy mechanism is very significant in regulating cell survival and homeostasis.^25, 26, 27, 28^ It has been widely studied that apoptotic signaling pathways can modulate autophagy, although autophagy can modulate apoptosis too.^25, 26, 29, 30^ Previous research has shown that autophagy is common in some malignant tumors and inhibition of autophagy increased chemotherapy sensitivity in some human tumors.^31, 32, 33^

At present, the data demonstrate that the effects of Apatinib on autophagy in osteosarcoma are rare. This study suggests that inhibition of autophagy promotes apoptosis. The presence of an autophagy inhibitor in Apatinib treatment is found to improve the therapeutic effect in osteosarcoma.

The possible mechanism that STAT3 induces apoptosis and autophagy were further studied. As an apoptosis-inhibiting gene, directly downstream of STAT3, BCL-2 is inhibited by Apatinib. In addition, knockdown of STAT3 aggravates Apatinib-induced BCL-2 inhibition. Previous reports suggest that STAT3 inactivation associates with altered cleaved-PARP expression and increased apoptosis.^34, 35^ Similarly, our research suggests that STAT3 mediated apoptosis by inhibiting the BCL-2 in osteosarcoma cells after Apatinib treatment.

More and more researches support presupposition that BCL-2 can inhibit autophagy by repressing Beclin-1.^36, 37, 38^ Accordingly, BCL-2 inhibition can increase the expression of Beclin-1 to induce autophagy.^39^ According to our research, BCL-2 downregulation by Apatinib or STAT3 siRNA increased the expression of Beclin-1, causing autophagy. The finding was validated by the overexpression of BCL-2 in osteosarcoma cells, which inhibited Apatinib-induced autophagy. In addition, it is also shown that Apatinib treatment of osteosarcoma xenografts led to decreased expression of BCL-2 and increase in cleaved-PARP, and suppressed growth of osteosarcoma *in vivo*.

Taken together, this study reveals for the first time that Apatinib exerts antitumor effects for osteosarcoma cells *in vitro* and *in vivo*. The benefits can be interpreted by VEGFR2-mediated STAT3 deactivation and the inhibition of BCL-2, and the target induces autophagy and apoptosis. Combined use of an autophagy inhibitor can enhance the antitumoral effect of Apatinib, which could be useful in the molecular targeted therapy of osteosarcoma. These marked findings expand our intelligence of the benefits and the clinical application of Apatinib.

## Materials and methods

### Clinical tissue specimen

In our research, 45 paraffin-embedded osteosarcoma pathologic specimens were gathered following the agreements authorized by the ethics committee of Peking University People’s Hospital. None of the patients received antitumor treatment before surgery. Clinical and histopathological data were registered in a patients’ retrospective review.

### Cell culture, reagents, colony formation assay and cell viability assay

KHOS and MG63 cells were obtained from American Type Culture Collection (ATCC, Manassas, VA, USA). These two cells were cultured in DMEM (Hyclone, Logan, UT, USA) with 10% fetal calf serum (Gibco, Grand Island, NY, USA) in a 37 °C humidified incubator in 5% CO_2_. All the experiments were conducted in the exponential phase of cell.

The following antibodies, anti-VEGFR2, anti-p-STAT3, anti-STAT3, anti-cyclin D1, anti-BCL-2, anti-Bax, anti-cleaved-PARP, anti-LC3, anti-p62 and anti-GAPDH, were obtained from Cell Signaling Technology (Beverly, MA, USA). CQ was obtained from Selleck (Houston, TX, USA).

The CCK8 (Dojindo Laboratories, Kumamoto, Japan) assay was used to evaluate the cell viability as described previously.^40^ The day before the experiment, the cells were seeded 5000 cells per well in 96-well plates. The cells were incubated with Apatinib (Hengrui, Jiangsu, China) at an indicated condition.

### Apoptosis analysis and cell cycle

For cell-cycle assay, cells were fixed with 70% ethanol at −20 °C overnight, and stained with propidium iodide. For cell apoptosis analysis, cells were stained with the Annexin V/FITC Kit (BD Biosciences, San Jose, CA, USA) according to the manufacturer’s explanations and analyzed by flow cytometry after Apatinib treatment as described previously.^41^

### Quantitative RT-PCR

The total RNA was extracted by Trizol (Invitrogen, Carlsbad, CA, USA). The reverse transcription was carried out as described previously.^21^ The qRT-PCR primers were purchased from RiboBio (Guangzhou, China). GAPDH were used as controls.

### Western blot analysis

Proteins were obtained from different types of cell lysates, and equal amounts of protein were added to 7.5–12.5% SDS-PAGE gels with the NuPAGE system (Invitrogen), and then the SDS-PAGE gels were transferred to PVDF membranes as mentioned previously.^42^

### Immunoprecipitation

An appropriate amount of antibody was added into the cell lysis solution, and then incubated at 4 °C for 3 h. Protein A agarose (Vigorous Biotechnology, Beijing, China; P007) was incubated for 1 h. The immune precipitates were washed three times using a lysis solution followed by elution with an SDS loading buffer. The eluent was subjected to western blot.

### Transmission electron microscopy

TEM was implemented as mentioned previously.^43^ At 48 h after Apatinib treatment, 0.25% trypsin was used to digest the cells, and then fixed with 1.5% glutaraldehyde. Sections (100 nm) were stained by uranyl acetate and lead citrate, and then detected by an electron transmission microscope (H-600; Hitachi, Tokyo, Japan).

### Immunohistochemistry

Immunohistochemistry was performed as mentioned previously.^44^ Sections were reacted with anti-VEGFR2, anti-p-STAT3 and anti-BCL-2 antibodies (1 : 100 dilution) and then stained with a rabbit serum instead of target antibody as a negative control. Cells showing positive staining on the membranes and in the nucleus and cytoplasm were figured in at least 10 typical fields (× 400) and the average percentage of positive cells was calculated. The specimens were evaluated by two independent pathologists who were unware of the clinical information.

### Immunofluorescence

Cells were spread on coverslips and incubated with Apatinib a concentration of 10 *μ*M for 48 h, then fixed cells were permeabilized with 0.1% Triton X-100 on ice, incubated with anti-LC3, and then finally incubated with anti-rabbit IgG conjugated with Dylight 488 (1 : 200) for 30 min at 37 °C. The coverslips were detected with a confocal microscopy (Zeiss, Baden-Wurttemberg, Germany) after washing three times with PBS.

### TUNEL assay

Apoptosis detection was identified using with *In Situ* Cell Death Detection Kit, POD (Roche Diagnostics, Mannheim, Germany). In short, after being fixed with 4% paraformaldehyde and blocked with 3% H_2_O_2_, the coverslips were washed with PBS two times and permeated with 0.1% Triton X-100 on ice for 2 min. Afterward, the coverslips were treated with TUNEL reagent at 37 °C for 1 h in a dark and wet box. After being washed three times, the slides were detected under a fluorescence microscopy (Olympus, Tokyo, Japan).

### Gene knockdown using siRNA

SiVEGFR2, siSTAT3, siBECN1 and control siRNAs were obtained from GenePharma (Suzhou, Jiangsu, China). Osteosarcoma cells were transfected with siRNA by Lipofectamine 3000 (Invitrogen). The cells were cultivated for 48 h for further experiments.

### Ectopic expression

The plasmid containing Bcl2 cDNA or negative control was transfected into KHOS cells with Lipofectamine 3000 (Invitrogen). The medium was replaced after 24 h incubation, and then the cells were treated with Apatinib.

### Tumor xenografts

A 4- to 6-week-old BALB/c nude mice (Vitalriver, Beijing, China) were subcutaneously injected in the right flank with 2 × 10^6^ KHOS cells. The mice were fed in specific pathogen-free conditions, and when a palpable mass developed, the mice were randomly divided into two sets and were administered DMSO or Apatinib 50 mg/kg orally daily for 30 days. The tumor was scaled every other day for 4 days. The tumor volume was counted by (length × width^2^/2). The mice were killed on the 13th day after the treatment. Tumor samples were prepared for western blot and IHC.

### Statistical analysis

The SPSS18.0 software (Chicago, IL, USA) was used for statistical analyses. Data were analyzed by one-way analysis of variance with the Bonferroni multiple comparison test. Comparison between two groups was performed using Student’s *t*-tests. Data were represented as mean±S.D. *P<*0.05 was regarded as statistically very significant.

## Publisher’s Note

Springer Nature remains neutral with regard to jurisdictional claims in published maps and institutional affiliations.

## Figures and Tables

**Table 1:** Relationship between VEGFR2 expression and clinical characters in osteosarcoma (*n*=45)

**Variables**	**Cases**	**VEGFR2 lower expression**		**VEGFR2 higher expression**		***P*****-value**
		***N***	**%**	***N***	**%**	
*Gender*						0.807
Male	24	10	55.6	14	51.9	
Female	21	8	44.4	13	48.1	
						
*Age at diagnosis (years)*						0.799
≤20	29	12	66.7	17	63.0	
>20	16	6	33.3	10	37.0	
						
*Tumor location*						0.435
Femur	18	7	38.8	11	40.7	
Tibia	12	5	27.8	7	25.9	
Humerus	7	3	16.7	4	14.9	
Others	8	3	16.7	5	18.5	
						
*Histological types*						0.382
Osteoblastic	24	10	55.6	14	51.9	
Chondroblastic	13	5	27.8	8	29.6	
Others	8	3	16.7	5	18.5	
						
*Enneking stage*						0.019
I+II	31	16	83.3	15	59.3	
III	14	2	16.7	12	40.7	

Abbreviation: VEGFR2, vascular endothelial growth factor receptor-2
